# Intraglomerular Monocyte/Macrophage Infiltration and Macrophage–Myofibroblast Transition during Diabetic Nephropathy Is Regulated by the A_2B_ Adenosine Receptor

**DOI:** 10.3390/cells9041051

**Published:** 2020-04-23

**Authors:** Ángelo Torres, Katherin Muñoz, Yessica Nahuelpán, Angelo-Paolo R. Saez, Pablo Mendoza, Claudia Jara, Claudio Cappelli, Raibel Suarez, Carlos Oyarzún, Claudia Quezada, Rody San Martín

**Affiliations:** Laboratorio de Patología Molecular, Instituto de Bioquímica y Microbiología, Universidad Austral de Chile, Valdivia 511-0566, Chile; katherin.v.munoz@hotmail.com (K.M.); yessica.nahuelpan@alumnos.uach.cl (Y.N.); angelo.riquelme@alumnos.uach.cl (A.-P.R.S.); pa.men2a@gmail.com (P.M.); claudia.jaracancino@gmail.com (C.J.); claudio.cappellileon@gmail.com (C.C.); raibelsua@gmail.com (R.S.); carlosoyarzun@uach.cl (C.O.); claudiaquezada@uach.cl (C.Q.)

**Keywords:** adenosine, A_2B_ adenosine receptor, diabetic kidney disease, glomerulosclerosis, macrophage–myofibroblast transition

## Abstract

Diabetic nephropathy (DN) is considered the main cause of kidney disease in which myofibroblasts lead to renal fibrosis. Macrophages were recently identified as the major source of myofibroblasts in a process known as macrophage–myofibroblast transition (MMT). Adenosine levels increase during DN and in vivo administration of MRS1754, an antagonist of the A_2B_ adenosine receptor (A_2B_AR), attenuated glomerular fibrosis (glomerulosclerosis). We aimed to investigate the association between A_2B_AR and MMT in glomerulosclerosis during DN. Kidneys/glomeruli of non-diabetic, diabetic, and MRS1754-treated diabetic (DM+MRS1754) rats were processed for histopathologic, transcriptomic, flow cytometry, and cellular in vitro analyses. Macrophages were used for in vitro cell migration/transmigration assays and MMT studies. In vivo MRS1754 treatment attenuated the clinical and histopathological signs of glomerulosclerosis in DN rats. Transcriptomic analysis demonstrated a decrease in chemokine-chemoattractants/cell-adhesion genes of monocytes/macrophages in DM+MRS1754 glomeruli. The number of intraglomerular infiltrated macrophages and MMT cells increased in diabetic rats. This was reverted by MRS1754 treatment. In vitro cell migration/transmigration decreased in macrophages treated with MRS1754. Human macrophages cultured with adenosine and/or TGF-β induced MMT, a process which was reduced by MRS1754. We concluded that pharmacologic blockade of A_2B_AR attenuated some clinical signs of renal dysfunction and glomerulosclerosis, and decreased intraglomerular macrophage infiltration and MMT in DN rats.

## 1. Introduction

Due to the increasing incidence rate of diabetes worldwide [1,2], a rise in the occurrence of diabetes-associated pathologies is also predicted [3,4,5]. Diabetic nephropathy (DN) is an important microvascular complication [6] and about 40% of diabetic patients develop DN [2], a condition that affects close to 50% of patients with chronic kidney disease (CKD) and end-stage renal disease (ESRD) [3,7]. During DN, patients lose glomerular function. This is clinically manifested by the appearance of proteins in the urine (proteinuria; an albumin excretion rate ≥300 mg/24 h per gram of creatinine) and/or a reduced glomerular filtration rate (GFR; below 60 mL/min/1.73 m^2^) [2]. In addition, people with DN who reach the stage of CKD [8] show an increase in the production of urine (polyuria), the appearance of glucose in the urine (glycosuria), and an increase in blood urea nitrogen (BUN) and serum creatinine [9]. Currently, management of DN patients involves the use of antihypertensive, antidyslipidemic, and antidiabetic agents, however these drugs have only shown a modest efficacy in slowing the evolution of the disease [10]. Regardless of the treatment used, the progression of DN leads to renal fibrosis [11], which irreversibly remodels the parenchyma tissue replacing it with extracellular matrix (ECM), thereby losing functionality [12]. Renal fibrosis predisposes patients to organ replacement therapies, such as hemodialysis and kidney transplantation, which means serious economic and societal costs for health systems [1,2,13,14,15]. The pathophysiological events that trigger renal fibrosis are still unknown, however, this process is orchestrated by myofibroblasts, cells which have the capacity to generate pro-fibrotic mediators and to deposit ECM in damaged tissue [11,16,17,18]. A characteristic feature of DN is glomerular fibrosis, known as glomerulosclerosis, which alters the filtering capacity of the glomeruli [19]. During glomerulosclerosis resident cells secrete pro-inflammatory factors such as tumor necrosis factor alpha (TNF-α), interleukin 1 beta (IL-1β), and transforming growth factor beta (TGF-β) [20,21] which promote ECM synthesis [22,23,24] and myofibroblast accumulation in the glomerulus [25,26,27,28,29]. Myofibroblasts have contractile properties, they express alpha-smooth muscle actin (α-SMA), intermediate filaments type II, and secrete ECM components such as collagen type I (Col1) and fibronectin 1 (Fn-1) [30] that contribute to tissue fibrosis. However, the origin of myofibroblasts in the renal glomeruli during DN is not entirely clear. Using experimental models of CKD it has been proposed that pericytes, resident fibroblasts of the kidney and bone marrow mononuclear cells (BM-MNCs), could undergo a mesenchymal transition process during renal fibrosis [27,28,29,30,31,32]. More recent studies have shown that the main mononuclear cell to infiltrate the kidney during CKD is the monocyte/macrophage (F4/80+, CD68+) [33,34,35]. Additionally, cytokines and molecules secreted by kidney-resident cells contribute to monocyte/macrophage infiltration in the tubule-interstitium and the glomerulus [11,36]. When monocytes/macrophages infiltrate renal tissue as a result of tissue damage, they transition to myofibroblasts (α-SMA+, Col1+, Fn-1+) in a process named macrophage–myofibroblast transition (MMT) [34,35,37]. Notably, a recent study showed that CD68+ cells contributed to approximately 50% of the total α-SMA+ (myofibroblast and fibrotic marker) cell population within the kidney of diabetic mice [38]. Therefore, the infiltration of monocytes/macrophages and the acquisition of a myofibroblastic phenotype represent the major contribution of cells which favor the development of renal fibrosis. In turn, among the pro-inflammatory and pro-fibrotic factors that promote CKD, the TGF-β signaling pathway was identified as the main mediator of MMT [30,35,39,40,41,42]. However, the mechanisms that regulate monocyte/macrophage infiltration and MMT during diabetic glomerulosclerosis have not been studied.

The adenosine nucleoside is another molecule that is deregulated during DN [43,44,45], observing an increase in its plasma bioavailability compared to the baseline levels observed in healthy or diabetic patients without DN [45]. The extracellular concentration of adenosine in DN oscillates in the micromolar order, levels at which it can activate its low affinity A_2B_ adenosine receptor (A_2B_AR), contributing to renal fibrosis [46,47]. Glomerular A_2B_AR activation produce increased vascular endothelial growth factor (VEGF) and TGF-β release [44,48], which favors DN progression [49,50]. In addition, in different models of renal fibrosis treatment, an A_2B_AR antagonist can reverse renal collagen accumulation and proteinuria [51]. A remarkable effect is observed when treating diabetic rats with MRS1754, a selective A_2B_AR antagonist; in parallel to the improvements observed in renal function, there is also a decrease in glomerular α-SMA expression [44].

Due to the benefits associated with in vivo A_2B_AR blockade in DN rats, we proposed that the decrease in glomerular fibrosis and the improvement in renal function are due to decreased infiltration and myofibroblastic transdifferentiation of monocytes/macrophages in the glomerulus. We observed that the in vivo antagonism of the A_2B_AR reduced some clinical and histopathological signs of glomerulosclerosis, and decreased intraglomerular monocyte/macrophage infiltration and MMT in DN rats.

## 2. Materials and Methods

### 2.1. Animals and Sample Collection

To induce diabetes mellitus (DM) one month old Sprague-Dawley male rats (200–250 g) were injected with streptozotocin (STZ; Merck, Darmstadt, Germany) in a single intravenous dose of 65 mg/kg [52]. DM was confirmed by glycemia between 300–500 mg/dL at one week post-inoculation of STZ. Non-diabetic (control; Ctrl) rats were inoculated with an equivalent volume of STZ vehicle (citrate buffer pH 4.5). A group of DM rats (four weeks post-STZ inoculation) were treated during eight weeks with MRS1754 (DM+MRS1754; 0.5 mg/kg/48 h intraperitoneal; Tocris Bioscience, Bristol, UK) [44] or an equivalent volume of MRS1754 vehicle (DM+Veh). Glycemia and weight were measured every week and to collect urine samples all animals were exposed to metabolic gale during 6 h each week. At week twelve post-STZ inoculation all rats were euthanized by an overdose of an inhalational anesthetic (isoflurane) and 1 mL of whole blood sample (abdominal aorta) was taken from each animal. Kidneys were removed and stored in chilled 1X phosphate buffered saline (PBS) until their use for glomerulus isolation or processing for to histological analysis. All animal procedures were performed according to the Guide for the Care and Use of Laboratory Animals, eighth edition (2011) (http://grants.nih.gov/grants/olaw/guide-for-the-care-and-use-of-laboratory-animals.pdf) and approved by the Institutional Committee on the Use of Live Animals in Research at the Universidad Austral de Chile (animals approval number 309/2018).

### 2.2. Glomerulus Isolation

Kidneys were washed in 1X PBS, chopped, and sieved through 212, 150, 106, and 75 μm meshes, respectively. The material was collected and centrifuged at 1500× *g* for 5 min at room temperature and corresponded to glomeruli (purity ≥ 90%) [52], which were used for transcriptomic analysis, flow cytometry, and in vitro rat macrophage migration assay.

### 2.3. Glycosuria and Proteinuria

Rat urine was recollected in metabolic cages for 6 h, then the urine volume was measured. Glucose (#1400106, Wiener Lab, Rosario, Argentina) and protein (#1690007, Wiener Lab, Rosario, Argentina) contents in urine were quantified using the autoanalyzer CM250 (Wiener Lab, Rosario, Argentina).

### 2.4. Serum Urea and Creatinine

Twelve weeks after STZ inoculation one mL of whole blood from each rat was centrifuged at 1200× *g* for 10 min. Serum was removed, then urea (#1810328. Wiener Lab, Rosario, Argentina) and creatinine (#1260360, Wiener Lab, Rosario, Argentina) were measured using the automatic analyzer HumaStar 200 (#16895, HUMAN Diagnostics, Wiesbaden, Germany).

### 2.5. Histological Analysis and Immunohistofluorescence

Rat kidneys derived from Ctrl, DM+Veh, and DM+MRS1754 rats were removed and fixed in 4% paraformaldehyde (PFA), paraffin embedded and 5 μm sections were mounted on silanized slides. Then slices were deparaffined with xylene and hydrated using decreasing concentrations of alcohols (96%, 90%, 70%, and 50% ethanol). Samples were stained with Periodic Acid-Schiff (PAS) and Masson’s Trichrome (MT) (Merck KGaA, Darmstadt, Germany). Images were analyzed by quantifying the percentage of positive staining (PAS: purple; MT: sky-blue and blue color) inside the glomeruli area using the color-deconvolution plugin and area fraction/area measurements using the software Image J (NIH, Bethesda, MD, USA). For immunohistofluorescence, antigen retrieval was carried out using a citrate buffer (10 mM sodium citrate, 0.05% Tween 20, pH 6.0) by heating it in the microwave every 5 min for half an hour, finally allowing it to cool for 30 min at room temperature. For blocking, 2.5% normal horse serum and 1% bovine serum albumin (BSA; blocking solution) were used for 30 min each, respectively. Immunodetections were performed using primary anti-CD68 (ab125212, Abcam, Cambridge, UK), anti-α-SMA (SC-130617, Santa Cruz Biotechnology, California, USA), and anti-nephrin (AF3159, R&D System, Minneapolis, MN, USA) antibodies in blocking solution overnight at 4 °C. Secondary antibody Alexa 488 and 568 (1:250 dilution; Thermo Fisher Scientific, Waltham, MA, USA) were incubated during 45 min and 4’,6-diamidino-2-phenylindole (DAPI) (300 nM; Thermo Fisher Scientific, Massachusetts, USA) for 10 min was used as a nuclei counterstain. To decrease tissue autofluorescence, 3% Sudan black B (*w*/*v* in 80% ethanol) stain was employed for 20 min. Finally, samples were washed in 1X PBS and mounted using a fluorescent mounting medium (S3023, Agilent-DAKO, Santa Clara, CA, USA). Images were captured using an epifluorescence microscope (Zeiss) and analyzed using the software Image J (NIH, Maryland, USA).

### 2.6. Transcriptomic Analysis

The RNA of glomeruli from DM+Veh and DM+MRS1754 rats was extracted using the commercial kit Nucleospin RNA II (Macherey-Nagel, Duren, Germany) following the instructions specified by the manufacturer. The quality of total RNA was measured with the fragment analyzer (Advanced Analytical Technologies), considering a RNA quality number (RQN) equal or superior to 8 for library preparation. The RNA-seq library was performed using the TruSeq RNA Sample Preparation Kit (Illumina) and its quantitation was performed by qPCR using the Library Quant Kit Illumina GA (KAPA) following the manufacturer’s instructions. Libraries were clustered on-board and sequenced to generate 125 bp paired-end reads using the high-throughput sequencing system HiSeq2500 (Illumina). Sequences were mapped to the rat genome (ensembl.org) and the number of read counts per gene were determined for each library using the feature counts function of the Rsubread R library. To determine differential expression based on raw counts we used the DEseq2 R library, and a *p*-adjusted value equal or less than 0.05 was considered statistically significant. Transcripts with statistical differences (*p* ≤ 0.05) were subjected to the Database for Annotation, Visualization and Integrated Discovery (DAVID) v6.8 and Kyoto Encyclopedia of Genes and Genomes (KEGG) platforms [53,54].

### 2.7. Human and Rat Peripheral Blood Mononuclear Cell (PBMCs) Isolation

To isolate human and rat peripheral blood mononuclear cells (PBMCs) a protocol by flotation using a low density iodixanol (OptiPrep^TM^, Merck KGaA, Darmstadt, Germany; see Application Sheet C05 for details) barrier was used. Briefly, 10 mL of human or rat whole blood were mixed with 2.7 mL of 40% (*w*/*v*) iodixanol. In a 15 mL conical tube, 5 mL of diluted blood were deposited, then using a syringe and metal cannula this was underplayed on a 5 mL density barrier (1.078 g/mL). A layer of 0.5 mL of Tricine-buffered saline (TBS; 0.85% NaCl, 10 mM Tricine-NaOH, pH 7.4) was added on top and centrifuged at 700× *g* for 20 min at 20 °C. A white band was removed, diluted in 2 mL TBS and centrifuged at 400× *g* for 10 min. The pellet was resuspended in Roswell Park Memorial Institute (RPMI) medium (Thermo Fisher Scientific, Massachusetts, USA) without serum and cultured in a T75 flask for 1 h. Then medium was replaced by RMPI–10% fetal bovine serum (FBS; Thermo Fisher Scientific, Massachusetts, USA) and adherent cells were cultured for 7 days until their use for in vitro assays. Finally the presence of macrophages in human and rat PBMCs was confirmed by CD68 and F4/80 marker expression.

### 2.8. Rat Macrophage Migration Assay

The in vitro macrophage chemoattractant effect of conditioned medium (CM) of glomeruli from Ctrl, DM+Veh, and DM+MRS1754 rats was evaluated using a polycarbonate (PC) Boyden chamber (8 μm pore). Briefly, ~350 glomeruli were incubated in 1 mL of F10 medium (Thermo Fisher Scientific, Massachusetts, USA) without serum for twelve hours. The medium was centrifuged at 700× *g* for 10 min and then supernatant was passed through a 0.4 μm filter and 24-well plates with PC transwell inserts (Corning^®^, New York, NY, USA) were used for rat macrophage migration assays. The bottom of the transwell inserts were coated overnight with 15 μg/mL of bovine fibronectin. A total of 1 × 10^5^ rat macrophages were seeded into the top of the transwell inserts and 650 μL of CM were added in the bottom of the well. Five percent FBS was used as a chemoattractant positive control. Twelve hours later macrophages in the bottom of the well were fixed with 70% ethanol for 10 min and stained using DAPI (300 nM) for 10 min. Cells were counted using 400× magnification in five different quadrants.

### 2.9. Human Macrophage Transmigration Assay

The CultreCoat^®^ 96-Well BME-Coated Cell Invasion Optimization Assay (cat# 3484-096-K, ©2010, Trevigen, Inc. Gaithersburg, MD, USA) was used to evaluate the in vitro transmigration of human macrophages. Briefly, 25,000 human macrophages diluted in 25 μL of RPMI medium were seeded into the top of a PC Boyden chamber with an 8 μm pore. Then, 150 μL of low glucose (LG; 5 mM D-glucose) or high glucose (HG; 25 mM D-glucose) medium was added to the bottom chamber with or without 5% FBS as the chemoattractant. Treatments (1 μM adenosine and/or 10 nM MRS1754) were added and macrophages were incubated at 37 °C in a CO_2_ incubator for 24 h. The number of cells that transmigrated was quantified through calcein AM incorporation and fluorescence was measured at 485 nm excitation and 520 nm emission.

### 2.10. Flow Cytometry Assay

An adaptation of the protocol described by Rubio-Navarro et al., [36] was employed. Rat glomeruli were incubated with 0.05% collagenase type I for 15 min at 37 °C in an orbital shaker. Digestion was stopped using 3X the volume of DMEM:F12–10% FBS and then centrifuged at 1000× *g* for 5 min. Pellets were resuspended and incubated in 500 µL of 3.7% PFA for 5 min at room temperature and then spun down at 700× *g* for 5 min. Sediments were incubated in 500 µL of rat serum for 45 min followed by primary antibody anti-CD68 (ab125212, Abcam, Cambridge, UK) and anti-α-SMA (SC-130617, Santa Cruz Biotechnology, California, USA) overnight at 4 °C. Secondary antibody Alexa 488 and 568 (1:250 dilution; Thermo Fisher Scientific, Massachusetts, USA) were incubated for 45 min and then cells were centrifuged at 700× *g* for 5 min. Finally, cells were analyzed by flow cytometry FACS Jazz (BD Biosciences, New York, NY, USA).

### 2.11. Immunocytofluorescence

Human macrophages were seeded in circular coverslips (diameter 18 mm) in 6-well culture plates. Cells were cultured in RMPI medium 0.5% FBS and treated with 1 µM adenosine (ADO; cat# A9251, Merck KGaA, Darmstadt, Germany), 10 ng/mL TGF-β1 (cat# 240-B, R&D systems, Minneapolis, MN, USA), and/or 10 nM MRS1754 every day for one week. Then macrophages were fixed with 3.7% PFA for 10 min and permeabilizated using 1X PBS–0.1% Triton X-100 for 10 min. Cells were blocked using 2.5% normal horse serum and 1% BSA (blocking solution) and then incubated with primary antibody anti-CD68 (ab125212, Abcam, Cambridge, UK), anti-F4/80 (ab111101, Abcam, Cambridge, UK), anti-α-SMA (SC-130617, Santa Cruz Biotechnology, California, USA) and anti-Collagen type I alpha-1 (Col1α1; SC-8784, Santa Cruz Biotechnology, California, USA) in blocking solution overnight at 4 °C. Secondary antibody Alexa 488 and 568 (1:250 dilution; Thermo Fisher Scientific, Massachusetts, USA) were incubated for 45 min and DAPI for 10 min was used as counterstain of nuclei. Finally, samples were washed in 1X PBS and mounted using a fluorescent mounting medium (S3023, Agilent-DAKO, Santa Clara, CA, USA). Images were captured using an epifluorescence microscope (Zeiss) and analyzed with Image J software (NIH, Maryland, USA).

### 2.12. RNA Isolation and RT-qPCR

Total RNA was isolated from human macrophages using Trizol^®^ reagent according to the manufacturer’s instructions (Invitrogen^®^, San Diego, CA, USA). Total RNA (1 µg) was used to synthesize cDNA using Moloney murine leukemia virus (M-MLV) reverse transcriptase (Promega^®^, Madison, WI, USA) and 25 mM Mix OligodT (15–17 mer; IDT^®^, San Jose, CA, USA). qPCRs were performed in a MX-3005 real-time PCR thermal cycler (Stratagene^®^, Santiago, Chile) using SYBR green Brilliant Blue III reagent (Agilent^®^, Santa Clara, CA, USA) with gene-specific primers targeting α-SMA, TGF-β, collagen type I alpha-2 (Col1α2), fibronectin 1 (Fn-1) and hypoxanthine phospho-ribosyl transferase-1 (HPRT-1) (listed in Table S1). HPRT-1 was employed as an internal control (normalizer) and dissociation curves were run to detect non-specific amplification. Relative gene expression levels were calculated after normalization with internal control HPRT-1 gene using the 2^−ΔΔCt^ method and expressed as the fold change compared to the control [55].

### 2.13. Statistical Analysis

Values are means ± standard deviation (SD), where n indicates the number of animals or times which the assay was performed. Comparisons between two or more groups were performed by means of the unpaired Student’s *t* test and two way ANOVA, respectively. If the ANOVA demonstrated a significant interaction between variables, post hoc analyses were performed by the multiple-comparison Bonferroni correction test. *p* < 0.05 was considered statistically significant.

## 3. Results

### 3.1. In Vivo MRS1754 Treatment Attenuates the Clinical Signs of Glomerular Injuries and Decreases Glomerular Fibrosis in Diabetic Rats

**Figure 1:**
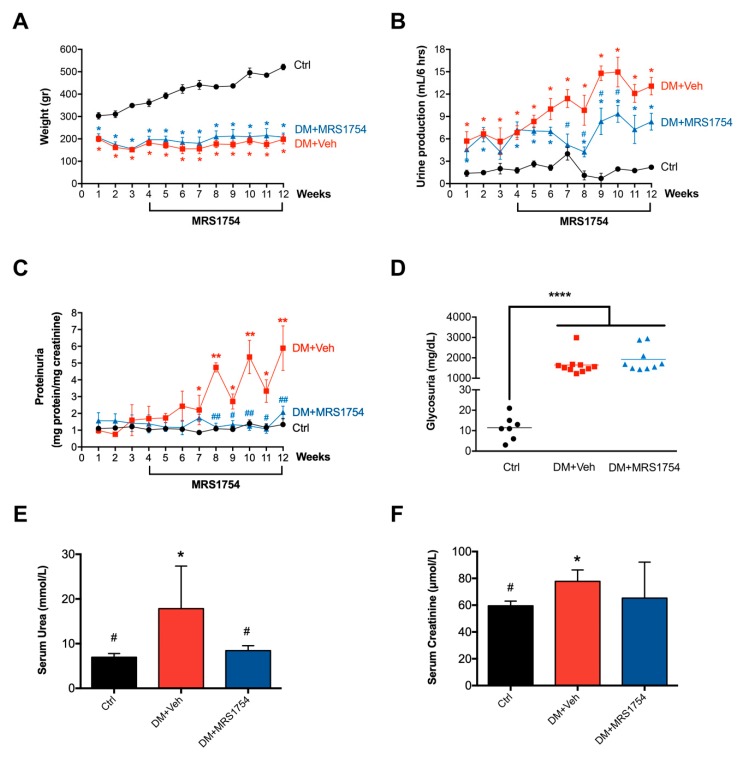
In vivo pharmacological blockade of A_2B_AR attenuates the clinical signs of glomerular injuries in diabetic nephropathy rats. Pathophysiological parameters of renal function were evaluated in non-diabetic (black; control (Ctrl)), diabetic vehicle-treated (red; DM+Veh) and diabetic MRS1754-treated (blue; DM+MRS1754) rats for twelve weeks post-streptozotocin (STZ) inoculation. Diabetic rats were treated with MRS1754 (0.5 mg/kg/48 h) for eight weeks, four weeks after STZ inoculation. (**A**) Weight (grams) was determined each week and urine was collected in a metabolic cage for 6 h to measure its (**B**) production (mL), (**C**) protein (mg protein/mg creatinine) and (**D**) glucose (mg/dL) content. Proteinuria was normalized to creatinine (mg protein/mg creatinine). (**E**) Urea (mmol/L), and (**F**) creatinine (μmol/L) were quantified in the serum at twelve weeks post-STZ inoculation. Glycosuria, serum concentration of urea, and creatinine were measured at twelve weeks after STZ inoculation. Graphs represent the mean ± S.D. * *p* < 0.05, ** *p* < 0.01, **** *p* < 0.001 versus Ctrl rats. ^#^ *p* < 0.05, ^##^ *p* < 0.01 DM+MRS1754 versus DM+Veh rats. n = 6.

One-month-old streptozotocin (STZ)-induced diabetic mellitus (DM) rats were treated with MRS1754 (DM+MRS1754), an antagonist of the A_2B_ adenosine receptor (A_2B_AR), for eight weeks. Pathophysiological parameters were measured each week, showing a decrease in weight gain from the first week post STZ inoculation (Figure 1A). Decreased renal function was evidenced by an increase in urine production (polyurea; Figure 1B) and the presence of protein (Figure 1C) and glucose (Figure 1D) in urine (proteinuria and glycosuria, respectively) in DM rats. Moreover, the serum concentration of urea and creatinine were measured at the twelfth week after STZ inoculation. High levels of blood urea nitrogen (BUN) and serum creatinine were observed in DM rats (Figure 1E,F). MRS1754 treatment attenuated polyuria, proteinuria, and serum urea but not the loss of body weight and glycosuria in DM rats (Figure 1A–F). Nevertheless, there were no differences in serum creatinine levels between non-diabetic (control = Ctrl) and DM+MRS1754 animals (Figure 1F).

**Figure 2:**
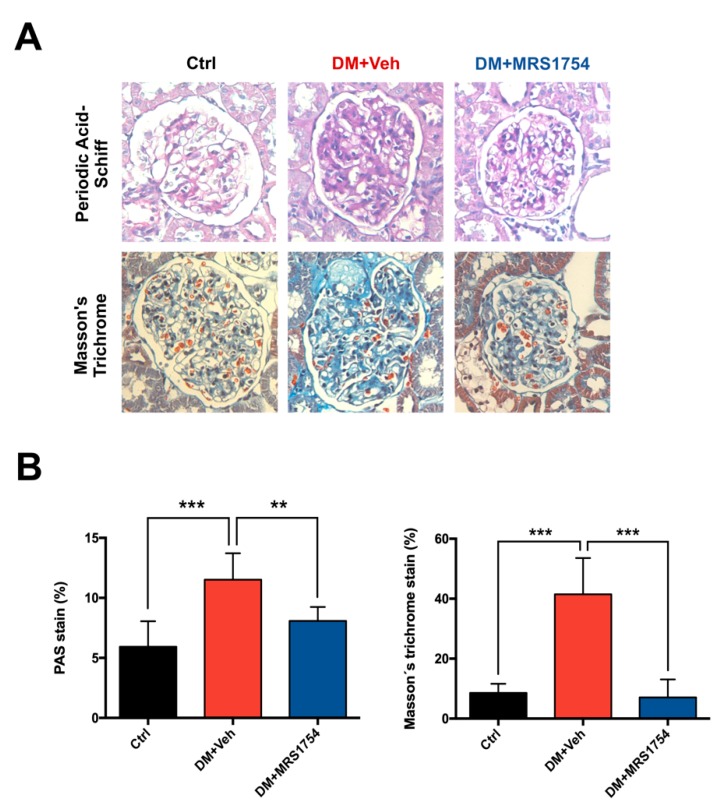
In vivo MRS1754 treatment decreases glomerular fibrosis in diabetic rats. (**A**) Periodic acid–Schiff (PAS) and Masson’s trichrome (MT) stains with its (**B**) quantification in kidneys of non-diabetic (black; Ctrl), diabetic vehicle-treated (red; DM+Veh) and diabetic MRS1754-treated (blue; DM+MRS1754) rats at week twelve post-STZ inoculation. Diabetic rats were treated with MRS1754 (0.5mg/kg/48 h i.p.) for eight weeks, four weeks after STZ inoculation. Graphs represent the percentage (mean ± S.D) of PAS or MT stained area in the glomeruli. ** *p* < 0.01, *** *p* < 0.005 between groups. n = 6. Magnification 400×.

Histopathological analysis of the percentage of the glomeruli area stained with periodic acid–Schiff (PAS) and Masson’s trichrome (MT) demonstrated an increase in extracellular matrix (ECM) accumulation in glomeruli of DM rats (11.51% ± 0.45 and 41.5% ± 5.40, respectively) which is a characteristic of glomerular sclerosis, glomerular hypertrophy, and mesangial expansion in DM rats (Figure 2). DM+MRS1754 rats showed less intraglomerular and periglomerular ECM accumulation than vehicle-treated DM (DM+Veh) rats, being 8.07 ± 0.45 % and 7.09 ± 1.04 % in PAS and MT stains, respectively (Figure 2). These results suggest that the in vivo antagonism of A_2B_AR alleviates some clinical and histopathological signs of glomerulosclerosis in DM rats.

### 3.2. In Vivo Antagonism of A_2B_AR in Diabetic Rats Decreases the Transcriptional Expression of Chemokine-Chemoattractant and Cell Adhesion Molecules for Monocytes/Macrophages in Ex Vivo Glomeruli

**Figure 3:**
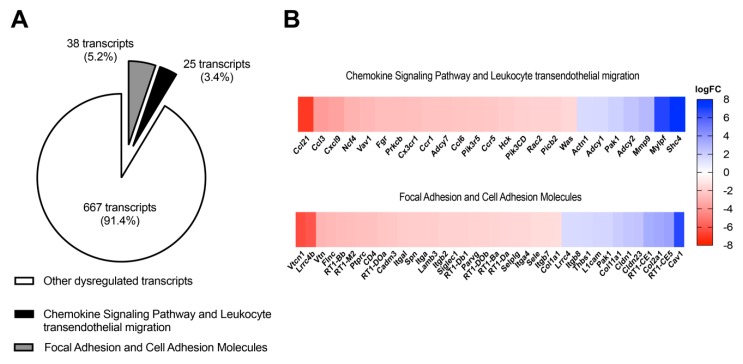
In vivo administration of MRS1754 reduces the transcriptional levels of genes related to chemokine/chemoattractant, cell adhesion, and maturation and function of monocyte/macrophage in the glomeruli of DN rats. (**A**) A total of 14,311 transcripts were analyzed by RNAseq, 730 of them were dysregulated and had a *p*-adjusted value equal or less than 0.05 in the glomeruli of MRS1754-treated DN rats. Thirty eight dysregulated transcripts were associated with focal adhesion and cell adhesion molecules (CAMs), and 25 transcripts related to the chemokine signaling pathway and leukocyte transendothelial migration were dysregulated. (**B**) Heatmap (logFC) of the top dysregulated transcripts related to the chemokine signaling pathway and leukocyte transendothelial migration and focal adhesion and CAMs in glomeruli of MRS1754-treated DN rats. Downregulated and upregulated genes in the heatmap are represented in red and blue, respectively.

Glomeruli of DM+Veh and DM+MRS1754 rats were isolated and processed for transcriptomic analysis. A total of 14,311 transcripts were analyzed by RNAseq and 730 of them were significantly dysregulated (*p* ≤ 0.05). A pathway data analysis using the Kyoto Encyclopedia of Genes and Genomes (KEGG) showed enrichment of dysregulated transcripts related to focal adhesion/cell adhesion molecules (*p* value: 1.3 × 10^−3^/3.6 × 10^−8^; total 38 transcripts) and the chemokine signaling pathway/leukocyte transendothelial migration (*p* value: 2.3 × 10^−4^/1.3 × 10^−4^; total 25 transcripts) in the glomeruli of DM+MRS1754 rats (Figure 3A). Other dysregulated pathways were related to natural killer-cell-mediated cytotoxicity, regulation of actin cytoskeleton, complement and coagulation cascades, and viral myocarditis (Table S2). Curiously members of the chemokine family (CCL3, CCL6, CCL21, CXCL9, CX3CR1, CCR1, and CCR5), cell adhesion molecules (SELE, SELPLG, ITGAM, ITGA4, ITGB2, and ITGAL), and maturation/function (NCF4, Vav1, FGR, PRKCb, ADCY7, HCK and Rac2) genes of monocyte/macrophage [56,57] presented decreased expression in the glomeruli of MRS1754-treated DM rats (Figure 3B, Tables S3 and S4). These data suggest that in vivo antagonism of A_2B_AR in the glomeruli could attenuate glomerular monocyte/macrophage infiltration in DN rats.

### 3.3. MRS1754 Treatment Decreases the Migration/Transmigration and Intraglomerular Monocyte/Macrophage Infiltration by Inhibiting the Chemoattractant Effect Induced by Diabetic Rat Glomeruli

**Figure 4:**
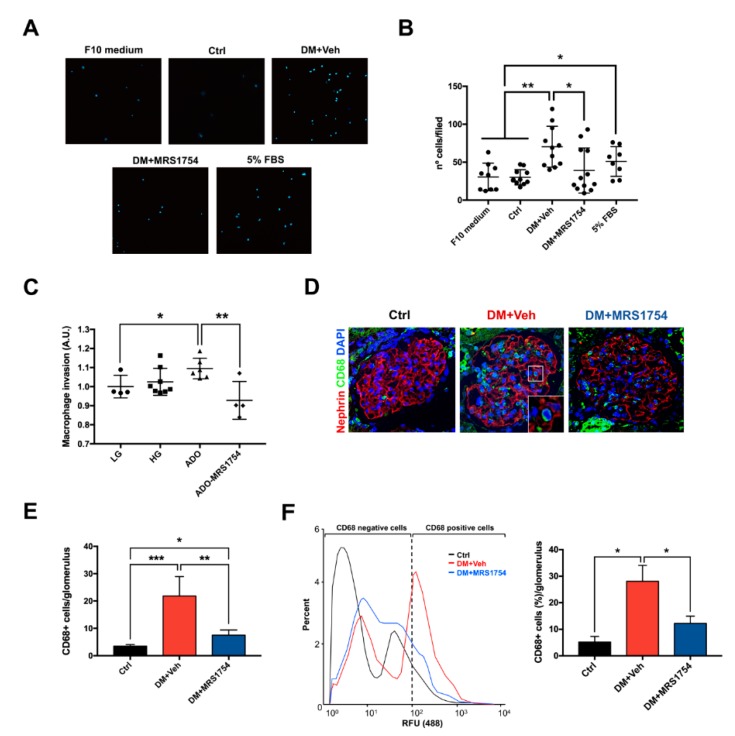
MRS1754 treatment decreases in vitro and in vivo macrophage cell migration and transmigration. (**A**) Migration of rat macrophages was evaluated for twelve hours in a transwell polycarbonate Boyden chamber under the chemoattractant effect of glomeruli-conditioned medium (CM) of non-diabetic (Ctrl), diabetic vehicle-treated (DM+Veh), and diabetic MRS1754-treated (DM+MRS1754) rats. F10 medium and 5% fetal bovine serum (FBS) were used as negative and positive controls, respectively, for macrophage cell migration. 4’,6-diamidino-2-phenylindole (DAPI) was used to stain the nuclei of macrophages. (**B**) Graphs represent the macrophage number per field (nº cells/filed) in (A)). (**C**) Cell transmigration (arbitrary unit = A.U.) of human macrophages was tested for twenty-four hours in transwell polycarbonate Boyden chamber coated with growth-factor-reduced basement membrane extract (BME) under the chemoattractant effect of 5% FBS and incubated in low D-glucose (LG; 5 mM D-glucose) or high D-glucose (HG; 25 mM D-glucose) medium. Additionally, the HG condition was supplemented with adenosine (ADO; 1 μM) and MRS1754 (10 nM). (**D**) Immunohistofluorescence of CD68 (macrophage marker; green color) and nephrin (podocyte marker; red) in the glomeruli of non-diabetic (black; Ctrl), diabetic (red; DM+Veh), and diabetic MRS1754-treated (blue; DM+MRS1754) rats at twelve weeks post-STZ inoculation. DAPI (blue) was used as a counterstain. The box at lower right corner of the middle image represents a magnification of the selected area. (**E**) Graphs represent quantification of CD68+ cells per glomerulus in (D). (**F**) Flow cytometry for the CD68 macrophage marker in collagenase-digested glomeruli of non-diabetic (black; Ctrl), diabetic (red; DM+Veh), and diabetic MRS1754-treated (blue; DM+MRS1754) rats at twelve weeks post-STZ inoculation. Histogram (left panel) and graph (right panel) of the percentage of CD68+ cells in rat glomeruli. Relative fluorescence units = RFU. Graphs represent the mean ± S.D. * *p* < 0.05, ** *p* < 0.01, *** *p* < 0.005 between groups. n = 4. Selected glomerulus area with a 400× magnification.

To evaluate the monocyte/macrophage chemoattractant effect of DM rat glomeruli we incubated rat monocyte/macrophages with the conditioned medium (CM) of rat glomeruli ex vivo which were cultured for 12 h. CM of DM+Veh rat glomeruli increased the migration of rat monocytes/macrophages by ~2-fold (70.36 ± 8.11 monocytes/macrophages per field) compared to non-diabetic (Ctrl) rat glomeruli (30.09 ± 3.01 monocytes/macrophages per field). Rat monocyte/macrophage migration was decreased when treated with CM of glomeruli isolated from DM+MRS1754 rats (39 ± 8.57 monocytes/macrophages per field) (Figure 4A,B). Because the glomeruli of diabetic rats generate high levels of extracellular adenosine [44] we performed an in vitro transmigration assay to determine the direct effect of adenosine and A_2B_AR on macrophage invasion. The adenosine stimulus increased in vitro transmigration of human macrophages by ~10%, which was reverted when using MRS1754, decreasing by up to ~15% compared to the adenosine condition (Figure 4C). To determine the role of A_2B_AR in in vivo intraglomerular monocyte/macrophage infiltration we performed immunohistofluorescence for CD68 (rat macrophage marker) in the renal tissue of Ctrl, DM+Veh and DM+MRS1754 rats. Twelve-week DM+Veh rats presented an increase in the number of CD68+ cells into the glomeruli by up to ~6.2-fold compared to Ctrl rats (21.83 ± 2.90 and 3.5 ± 0.28 macrophages per glomerulus, respectively). Intraglomerular CD68+ cells decreased by 7.5 ± 0.95 per glomerulus in DM+MRS1754 rats (Figure 4D,E). To corroborate intraglomerular macrophage infiltration at week twelve post-STZ inoculation, the glomeruli of Ctrl, DM+Veh, and DM+MRS1754 rats were exposed to collagenase digestion followed by flow cytometry studies. The abundance of CD68+ cells in Ctrl rats was 5.19 ± 1.23% and in DM+Veh rats this increased by up to 28.08 ± 2.45% (Figure 4F). In vivo antagonism of A_2B_AR decreased the abundance of CD68+ cells by up to 12.22 ± 0.96% in DM rats (Figure 4F). These results demonstrate that MRS1754 treatment decreases in vitro macrophage migration/transmigration and in vivo intraglomerular infiltration of monocytes/macrophages in DM rats.

### 3.4. A_2B_AR Blockade Decreases the Intraglomerular Macrophage–Myofibroblast Transition in Diabetic Rats

**Figure 5:**
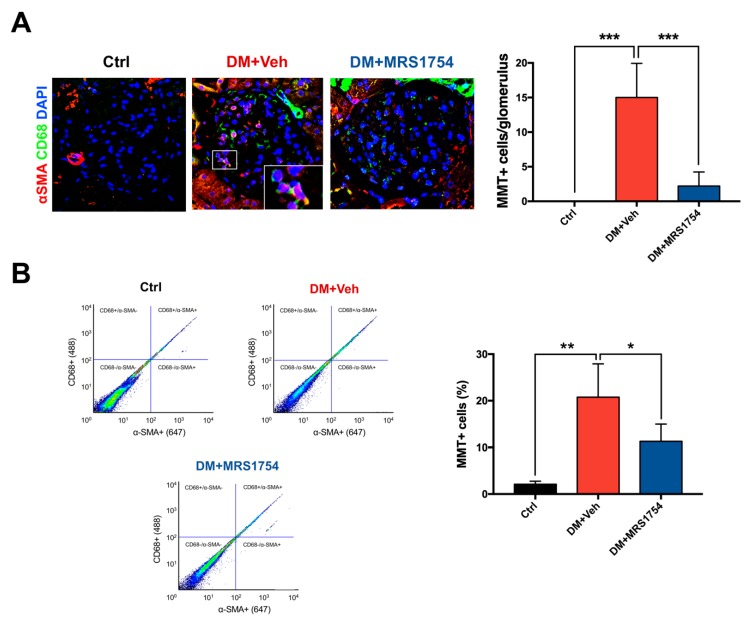
A_2B_AR antagonism decreases in vivo intraglomerular macrophage–myofibroblast transition in diabetic nephropathy rats. (**A**) Immunohistofluorescence of CD68 (macrophage marker; green color) and α-SMA (myofibroblast marker; red color) in glomeruli of non-diabetic (black; Ctrl), diabetic (red; DM+Veh), and diabetic MRS1754-treated (blue; DM+MRS1754) rats at twelve week post-STZ inoculation. DAPI (blue) was used as a counterstain. The box at the lower right corner of the middle image represents a magnification of the selected area. Graphs (right panel) represent quantification of MMT+ (CD68+/α-SMA+) cells per glomerulus. (**B**) Flow cytometry for CD68 and α-SMA in collagenase-digested glomeruli of non-diabetic (black; Ctrl), diabetic (red; DM+Veh), and diabetic MRS1754-treated (blue; DM+MRS1754) rats at twelve weeks post-STZ inoculation. Flow cytometry dot plots (left panel) and graph (right panel) represent the percentage of MMT+ cells in rat glomeruli. Dot plots show the cell abundance in different colors, being green/yellow-red and blue/sky-blue colors high and low number of cells, respectively. Graphs represent the mean ± S.D. * *p* < 0.05, ** *p* < 0.01, *** *p* < 0.005 between groups. n = 4. Selected glomerulus area with a 400× magnification.

Macrophage–myofibroblast transition (MMT) has been reported as the main source of myofibroblasts in in vivo models of fibrotic kidney disease [34,38]. Since MRS1754 treatment decreases intraglomerular monocyte/macrophage infiltration, we investigated the effect of A_2B_AR blockade on MMT within the glomerulus. Immunohistofluorescence for CD68 (macrophage marker) and alpha-smooth muscle actin (α-SMA; myofibroblast marker) was performed in renal tissue of Ctrl, DM+Veh, and DM+MRS1754 rats. MMT+ was determined by quantification of double positive CD68 and α-SMA (CD68+/α-SMA+) cells. Inside the glomeruli of DM+Veh rats the number of MMT+ cells increased 15-fold (15 ± 2.21 cells per glomerulus) compared to Ctrl rats in which non double positive cells were observed (Figure 5A). In vivo treatment with MRS1754 decreased the number of MMT+ cells up to 2.2 ± 0.91 per glomerulus (Figure 5A).

However, we also observed macrophages that were negative for the myofibroblast marker (CD68+/α-SMA- cells) and myofibroblasts that were negative for the macrophage marker (CD68-/α-SMA+ cells). To quantify these cells we performed a flow cytometry analysis for CD68 and α-SMA markers in cell suspensions of collagenase-digested glomeruli from Ctrl, DM+Veh, and DM+MRS1754 rats. Over ~99% of α-SMA+ cells were also positive for the CD68 macrophage marker in the glomeruli of Ctrl, DM+Veh, and DM+MRS1754 rats. The percentage of macrophages positive for α-SMA+ was ~77% and ~98% in the glomeruli of Ctrl and DM+Veh rats, respectively. Further, MRS1754 treatment in diabetic rats decreased this percentage (~71%) (Table S5). The percentage of MMT cells in the glomeruli of Ctrl rats was 2.1 ± 0.37%, ~10-fold less than glomeruli of DM+Veh rats (20.77 ± 3.57 %) (Figure 5B). In vivo A_2B_AR blockade decreased the percentage of MMT cells by up to 11.3 ± 1.84%, half the value in the glomeruli of DM rats (Figure 5B).

### 3.5. In Vitro MRS1754 Treatment Reduces Macrophage to Myofibroblast-Like Morphology and MMT Markers in Human Macrophages

**Figure 6:**
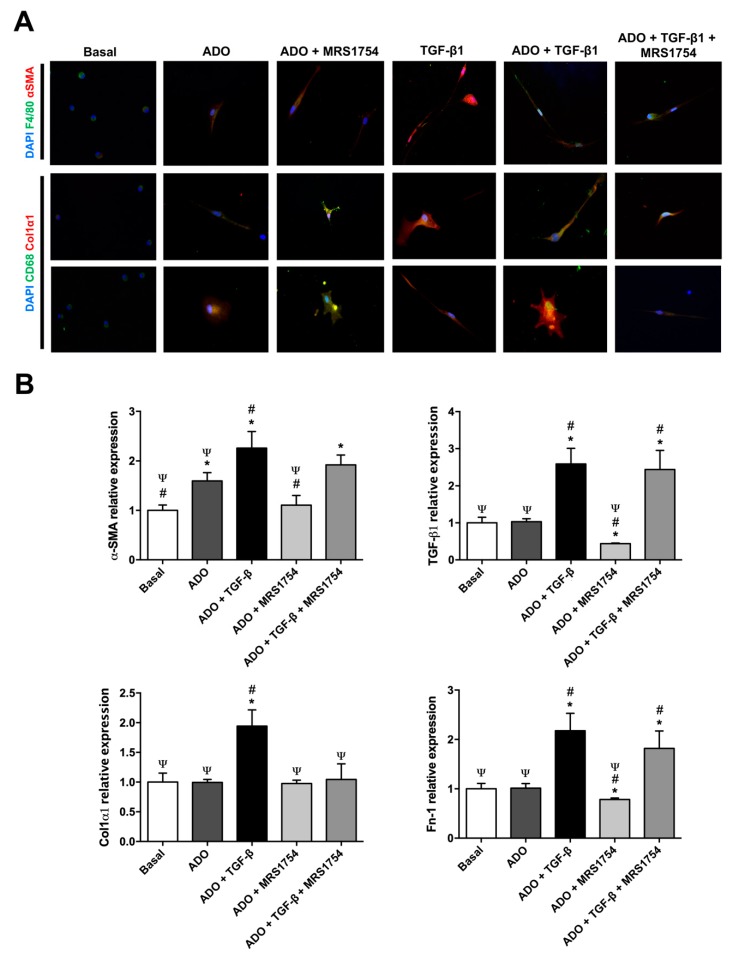
In vitro blockade of A_2B_AR decreased myofibroblast marker expression in macrophages. (**A**) Immunocytofluorescence of macrophage (F4/80 and CD68; green) and myofibroblast (α-SMA and Col1α1; red) markers in human macrophages cultured in Roswell Park Memorial Institute (RPMI) high D-glucose medium 0.5% FBS (basal) treated with 1 µM adenosine (ADO), 10 ng/mL TGF-β1, and 10 nM MRS1754 for seven days. DAPI (blue) was used as a counterstain. Cells selected from the image captured with a 400× magnification. (**B**) mRNA expression of myofibroblast markers α-SMA, TGF-β1, Col1α2, and Fn-1 by RT-qPCR in human macrophages cultured under basal condition, adenosine (ADO), TGF-β1, and MRS1754 for seven days. HPRT mRNA expression was used for normalization. Graphs represent the mean ± S.D. * *p* < 0.05 versus basal; # *p* < 0.05 versus ADO; Ψ *p* < 0.05 versus ADO + TGF-β1. n = 3.

Since TGF-β stimuli can induce MMT [30,35,39,40,41,42] we evaluated the effect of adenosine and/or the blockade of A_2B_AR activation on human macrophages incubated with TGF-β for seven days. A spherical shape was observed in peripheral blood human macrophages which were positive by immunocytofluorescence (ICF) for macrophage markers F4/80 and CD68 (Figure 6A). These macrophages were incubated with adenosine and showed elongated or circular spreading shapes, and also expressed the myofibroblast markers α-SMA and Col1α1 (Figure 6A). TGF-β and adenosine/TGF-β treatments induced an evident elongated and thin shape with large cytoplasmic extensions and high expression of MMT markers; conditions which were partially reverted using MRS1754 (Figure 6A). mRNA expression of myofibroblast markers (α-SMA, TGF-β, Col1α2, and Fn-1) was evaluated by RT-qPCR in human macrophages treated with adenosine, MRS1754, and/or TGF-β for seven days. Adenosine stimuli increased α-SMA expression by ~1.6-fold and in vitro A_2B_AR blockade reverted this effect (Figure 6B). Incubation with adenosine/TGF-β increased transcriptional expression of α-SMA, TGF-β, Col1α2, and Fn-1 compared to basal (1.25, 1.6, 0.94, and 1.17, respectively) and adenosine (0.66, 1.6, 0.94, and 1.17, respectively) conditions (Figure 6B). MRS1754 treatment abolished the expression of Col1α1 induced by adenosine/TGF-β (Figure 6B). These data propose that A_2B_AR antagonism partially abrogates MMT induced by adenosine and/or TGF-β in human macrophages.

## 4. Discussion

We demonstrate that monocyte/macrophage infiltration and MMT promoted by diabetes in renal glomerulus is attenuated by treatment with an A_2B_AR antagonist, which correlates with an improvement in some clinical parameters of glomerular function and kidney histology preservation. In the present study, we used a STZ-induced DN model in rats, which resembles the human condition in a clinical and pathophysiological manner [58]. Diabetic rats showed a decrease in their weight gain and an increase in urine production, proteinuria, glycosuria, and azotemia (increased BUN and creatinine). In vivo treatment with a selective A_2B_AR antagonist (MRS1754; dose of 0.5 mg/kg/48 h for 56 days) managed to decrease the polyurea, proteinuria, and serum urea levels; nevertheless, MRS1754-treated DM rats did not show improvements in glycemia, weight gain, glycosuria, or serum creatinine. These data indicate that the effect of in vivo treatment with MRS1754 in DN rats does not reverse the diabetic condition but does relieve some clinical signs related to glomerular damage. STZ-induced DN in rats is a model that better resembles human diabetic glomerulopathy and parallels the remarkable increase of A_2B_AR at the glomerulus, as detected histologically in patients [43]. Thus, Cárdenas et al. demonstrated decreased proteinuria in diabetic rats inoculated with MRS1754 at a dose of 0.2 and 1 mg/kg/48 h for 14 days, reversing glomerulopathy [44]. However, other beneficial effects mediated by MRS1754 such as regulation of polyuria are not clearly understood, but it does suggest decreased glomerular filtration rate and sodium excretion at the collecting duct under pharmacological blockade of A_2B_AR [59,60]. Altogether, our findings highlight the safety of this treatment as no adverse effects were observed.

During DN, patients have a progressive loss of glomerular function, which has been attributed to a decrease in the number of podocytes (cells responsible for the glomerular ultrafiltration process) and the excessive ECM deposition at the glomerular level (glomerular fibrosis or glomerulosclerosis) [61,62]. In this study, we evaluated the degree of glomerular fibrosis using periodic acid-Schiff and Masson’s trichrome stains, showing an evident accumulation of glycoproteins and collagen in the glomeruli of diabetic rats. These results are histopathologically compatible with glomerular damage associated with diabetes, in which the glomerulus responds to injury by synthesizing and secreting ECM components (such as collagen, proteoglycans, and glycoproteins) thereby promoting the development of glomerulosclerosis [63]. Another research group recently reported that the pharmacological blockade of A_2B_AR using PSB1115 (200 µg/d) for 10 days prevents renal fibrosis in a mouse unilateral ureteral obstruction (UUO) model [51]. In our model, we observed that the use of MRS1754, despite being another model of renal fibrosis, shows a similar effect supporting the role of A_2B_AR in the development of glomerulosclerosis. In the study by Cardenas et al., the expression of α-SMA at the glomerular level was evaluated by immunohistochemistry as a fibrosis marker. A decrease in α-SMA staining was observed when using MRS1754 in doses of 1 mg/kg/48 h for 2 weeks, which was not appreciable using a dose of 0.2 mg/kg/48 h [44]. Another study performed by Xia’s group showed that high extracellular levels of adenosine and the activation of its A_2B_ receptor mediate the production of IL-6 and thereby promote renal fibrosis during CKD [64].

In recent years the role of immune cells in the renal fibrosis process has become very relevant, especially monocyte/macrophage function [65,66,67]. Initially during renal damage, macrophages can acquire a phenotype known as M1 (iNOS+/CD80+/CCR7+) which favors a pro-inflammatory microenvironment [66]. On the other hand, as the kidney disease progresses, these macrophages polarize to M2 (CD86-/CD163+/CD206+) which have an anti-inflammatory role promoting the healing of damaged tissue and are therefore considered pro-fibrotic [66,68]. This is why macrophage infiltration has been studied in different glomerular pathologies, proposing that glomerulus cells deliver chemoattractant chemokines which also promote the expression of cell adhesion molecules in macrophages [69,70,71,72]. One of these chemokines called MCP-1 (Monocyte chemoattractant protein-1) or CCL2 (C-C Motif Chemokine Ligand 2), has been described as the main chemoattractant mediator of monocytes/macrophages during renal inflammation, and is also related to fibrosis progression in different pathologies including DN [73,74].

The role of the glomerulus and extracellular adenosine to date have not been explored in the process of glomerular monocyte/macrophage infiltration during DN. In the transcriptional analysis of glomeruli of diabetic rats treated in vivo with MRS1754, a decrease in the expression of members of the chemokine family, cell adhesion molecules, and maturation/function of monocyte/macrophage related genes was observed [56,57]. Among these chemokines CCL21 (C-C Motif Chemokine Ligand 21) shows the most evident decrease compared to the diabetic glomeruli of untreated rats. This chemokine is widely known for its role in the homing of antigen-presenting cells and T lymphocytes in lymphoid organs [75]. It also has an important role in the infiltration of monocytes/macrophages at the renal level, and even regulates the production of CCL2 [76]. CCL21 is capable of activating the C-C chemokine receptor type 7 (CCR7) present in M1 macrophages showing a greater chemoattractant effect than the CCL2 chemokine [77]. In turn, inhibition of the CCL21/CCR7 axis decreases fibrosis at the renal level by almost 50% [78,79]. CCL21 secretion at the glomerular level has been demonstrated in mesangial cells, where this chemokine would participate in the early stages of renal inflammation as a protective agent [80,81]. On the other hand, the non-canonical activation of NFκB can promote the expression of CCL21 in podocytes; its expression being related to the progression of renal disease and proteinuria in rats [82]. This could explain the lower chemoattractant effect in vitro induced by the CM of diabetic rat glomeruli treated in vivo with MRS1754, a result that also corresponded to a lower in vivo intraglomerular infiltration of macrophages (CD68+), observed by immunohistofluorescence and flow cytometry in the glomeruli of diabetic rats treated with MRS1754. Similar results were observed by Xie et al., in a mouse UUO model using PSB1115, in which A_2B_AR antagonism decreased the expression of CCL2 and RANTES (Regulated upon Activation, Normal T cell Expressed, and Secreted) chemokines, and inhibited the infiltration and activation of M1 macrophages [51]. These results reveal the role that A_2B_AR plays at the glomerular level in the process of monocyte/macrophage infiltration during glomerulosclerosis in the DN. On the other hand, we evaluated the direct in vitro effect of adenosine and A_2B_AR on the ability of human macrophage transmigration and determined that the antagonism of this receptor is capable of diminishing this process. In this way, in vivo treatment with MRS1754 could exert its effect both at the glomerular level and directly on monocytes/macrophages, decreasing its renal infiltration during DN.

Previous studies show that inflammatory macrophages can transdifferentiate into myofibroblasts at the tubular level during renal fibrosis in a process known as MMT [34] which is orchestrated by the activation of the TGF-β1/smad3 axis [38]. The differentiation and activation of myofibroblasts is recognized as a central event in the pathogenesis of fibrosis and kidney disease due to excessive deposition and accumulation of ECM; a condition that also triggers the functional collapse of the kidney [83]. However, the origin of this renal population of myofibroblasts is not yet clear [27,84] and there are also few studies that evaluate myofibroblastic transdifferentiation at the intraglomerular level [85,86]. Additionally, it is unclear if A_2B_AR has any effect on the accumulation of myofibroblasts at the renal level or its implication in the MMT process. In the present study, MRS1754 was used. This had previously demonstrated an anti-fibrotic effect by decreasing the expression of intraglomerular α-SMA (myofibroblast marker) in STZ-induced diabetic rats [44]. That is why we assessed whether the population of monocytes/macrophages that was detected by infiltrating the glomeruli of diabetic rats corresponded to macrophages transdifferentiated into myofibroblasts (CD68+/α-SMA+). For this, immunohistofluorescence of CD68 and α-SMA was performed in kidney sections, detecting an intraglomerular increase in the positive signal for both markers in the glomeruli of diabetic rats, a signal that decreased in the glomeruli of rats treated with MRS1754, although it did not reach the basal levels of healthy non-diabetic rats. As other authors have reported, colocalization of the markers was observed not only within the glomerulus but also outside it [38]. The results of intraglomerular MMT by immunohistofluorescence complemented those obtained by flow cytometry of glomeruli digested with collagenase in which the population of cells undergoing MMT (CD68+/α-SMA+) was quantified. The results show a similar trend to that observed by immunohistofluorescence, increasing the percentage of MMT+ cells within the glomeruli of diabetic rats; a condition that was reversed by antagonizing A_2B_AR in vivo. However, it was also possible to detect the presence of a population of macrophages (CD68+) that were negative for α-SMA, possibly due to macrophages that had not yet transdifferentiated into myofibroblasts, as well as a population of myofibroblasts (α-SMA+) that were negative for CD68. These cells (CD68-/α-SMA+) may correspond to myofibroblasts from other cell lines such as kidney-resident fibroblasts, cells of hematopoietic origin, tubular epithelial cells, pericytes or infiltrated leukocytes [26,28,87,88]. Our results show that monocytes/macrophages that infiltrate at the glomerular level have the ability to transdifferentiate into myofibroblasts that express α-SMA in a diabetic context, which could be mediated by A_2B_AR activation. This may be due to the fact that A_2B_AR activation favors the secretion of TGF-β1 in glomeruli under a diabetic context [48] which would trigger the MMT event at the glomerular level. Activation of the TGF-β1/smad3 axis is considered the main regulator of renal fibrosis as it promotes myofibroblastic transdifferentiation of different cell types including M2 macrophages [34,89,90]. However, the direct mechanism by which adenosine promotes glomerular fibrosis is not yet clear since it has not been studied whether adenosine or the activation of its A_2B_ receptor directly participate in MMT. This is why the phenotypic (F4/80+, CD68+, α-SMA+, and Col1α1+) and morphological effects induced by treatment with adenosine, MRS1754, and/or TGF-β on MMT were evaluated in vitro by immunocytofluorescence in human macrophages. Macrophages in basal culture conditions expressed the surface markers CD68+ and F4/80+, and also presented a spherical morphology with few cytoplasmic expansions which corresponded to their typical characteristics under in vitro culture conditions [91]. When macrophages were incubated with adenosine, TGF-β, and adenosine/TGF-β we detected the expression of myofibroblast markers α-SMA and Col1α1. In addition, macrophages acquired an elongated or circular cytoplasmic morphology with radial (star-shape) extensions that matched the typical phenotypic characteristics of myofibroblasts, where α-SMA expression reflects an increase in contractile activity that would explain the prolonged extensions in its cytoplasm and the formation of focal adhesions and/or stress fibers [92,93].

Finally, in macrophages incubated with adenosine/MRS1754 and adenosine/TGF-β/MRS1754 a decrease in the intensity of myofibroblast markers and a reduction in cytoplasmic expansions were observed. To confirm that the morphological changes corresponded to myofibroblast transdifferentiation, an analysis of myofibroblast marker transcript expression (α-SMA, TGF-β, Col1α2, and Fn-1) was performed in human macrophages treated with adenosine, MRS1754, and/or TGF-β. Since differentiation of myofibroblasts is described as an event that involves multiple factors [94], it is important to consider the contractile potential granted by components such as α-SMA, collagen fibers, and fibronectin that make up the ECM [95,96]. Transcript measurements corroborate the increase in relative α-SMA expression when treating macrophages with adenosine and that this was reversed by antagonizing A_2B_AR. On the other hand, when macrophages were incubated with adenosine/TGF-β the effect was more noticeable and generalized, presenting increased expression of the four myofibroblast markers evaluated; however, when macrophages were treated with MRS1754 only a decrease in Col1α2 expression was observed. These results reveal that high levels of adenosine would allow A_2B_ receptor activation which would then regulate the in vitro transdifferentiation of macrophages to myofibroblasts. In a study by Liu et al., it was also demonstrated that extracellular accumulation of adenosine promotes the transition of fibroblasts to myofibroblasts by activating the A_2B_AR/TGF-β1/Fstl1 signaling pathway in a pulmonary fibrosis model in mice [97]. We concluded that pharmacologic blockade of A_2B_AR could be an alternative for attenuating diabetic nephropathy symptoms by affecting intraglomerular monocyte/macrophage infiltration and MMT that direct glomerular fibrosis.

## Supplementary Materials

The following are available online at https://www.mdpi.com/2073-4409/9/4/1051/s1, Table S1: List of primers sequence used for RT-qPCR; Table S2: List of the top dysregulated pathways analyzed using the Kyoto Encyclopedia of Genes and Genomes (KEGG) in glomeruli of MRS1754-treated DN rats; Table S3: List of the top Focal Adhesion and Cell Adhesion Molecules (CAMs) dysregulated transcripts in glomeruli of MRS1754-treated DN rats; Table S4: List of the top Chemokine Signaling Pathway and Leukocyte transendothelial migration dysregulated transcripts in glomeruli of MRS1754-treated DN rats; Table S5: Flow cytometry analysis of mean percent of Macrophages (CD68+) or Myofibroblasts (α-SMA+) positives and negatives for α-SMA or CD68 markers in the glomeruli of Ctrl, DM+Veh and DM+MR1754 rats.
