# Supplementary material for: Intraglomerular Monocyte/Macrophage Infiltration and Macrophage–Myofibroblast Transition during Diabetic Nephropathy Is Regulated by the A2B Adenosine Receptor
Source: Cells. 2020 Apr 23;9(4):1051. doi: 10.3390/cells9041051 (PMC7226348; doi:10.3390/cells9041051)
Supplement: Supplementary file 1 [file cells-09-01051-s001.zip › Supplementary table 1.pdf]

Supplementary table 1. List of primers sequence used for RT-qPCR.

Target	Primers sequence
Fn-1 mRNA	Fw: AACAAACACTAATGTTAATTGCCCA
	Rv: AGAGACATGCTTGTTCCTCTGG
TGF-β1 mRNA	Fw: GGAAATTGAGGGCTTTCGCC
	Rv: CCGGTAGTGAACCCGTTGAT
Col1α2 mRNA	Fw: CAAGGCATTCGTGGCGATA
	Rv: ACCATGGTGACCAGCGATAC
α-SMA mRNA	Fw: CCGGGACTAAGACGGGAATC
	Rv: TTGTCACACACCAAGGCAGT
HPRT-1 mRNA	Fw: CCCTGGCGTCGTGATTAGTG
	Rv: CACCCTTTCCAAATCCTCAGC
