# Supplementary material for: Intraglomerular Monocyte/Macrophage Infiltration and Macrophage–Myofibroblast Transition during Diabetic Nephropathy Is Regulated by the A2B Adenosine Receptor
Source: Cells. 2020 Apr 23;9(4):1051. doi: 10.3390/cells9041051 (PMC7226348; doi:10.3390/cells9041051)
Supplement: Supplementary file 1 [file cells-09-01051-s001.zip › Supplementary table 2.pdf]

Supplementary table 2. List of the top dysregulated pathways analyzed using the Kyoto Encyclopedia of Genes and Genomes (KEGG) in glomeruli of MRS1754-treated DN rats.

**PATHWAY**	**Dysregulated transcripts**	***p*-value**
Cell adhesion molecules (CAMs)	26	3.6E-8
Chemokine signaling pathway	21	2.3E-4
Focal adhesion	21	1.3E-3
Natural killer cell mediated cytotoxicity	20	2.6E-7
Regulation of actin cytoskeleton	18	2.7E-2
Complement and coagulation cascades	17	1.6E-7
Viral myocarditis	17	3.6E-6
Leukocyte transendothelial migration	17	1.3E-4
Systemic lupus erythematosus	16	2.5E-5
Hematopoietic cell lineage	13	3.6E-4
Antigen processing and presentation	13	1.1E-3
Fc gamma R-mediated phagocytosis	12	3.5E-3
Vascular smooth muscle contraction	12	2.2E-2
Axon quidance	12	4.6E-2
B cell receptor signaling pathway	11	3.3E-3
Toll-like receptor signaling pathway	11	1.2E-2
Type I diabetes mellitus	10	2.3E-3
