# Supplementary material for: Intraglomerular Monocyte/Macrophage Infiltration and Macrophage–Myofibroblast Transition during Diabetic Nephropathy Is Regulated by the A2B Adenosine Receptor
Source: Cells. 2020 Apr 23;9(4):1051. doi: 10.3390/cells9041051 (PMC7226348; doi:10.3390/cells9041051)
Supplement: Supplementary file 1 [file cells-09-01051-s001.zip › Supplementary table 3 and 4.pdf]

Supplementary table 3. List of the top Focal Adhesion and Cell Adhesion Molecules (CAMs) dysregulated transcripts in glomeruli of MRS1754-treated DN rats. Downregulated and upregulated genes are represented in red and blue respectively.

GENBANK_ACCESSION	GENE NAME	logFC	*p*-value
NM_001024244	V-set domain containing T cell activation inhibitor 1(Vtcn1)	-6,42	0,01
NM_001271081	Leucine rich repeat containing 4B(Lrrc4b)	-6,10	0,02
NM_019156	Vitronectin(Vtn)	-2,89	0,02
NM_001191862	Filamin C(Flnc)	-2,70	0,01
NM_001004084	RT1 class II, locus Bb(RT1-Bb)	-2,61	0,00
NM_001001717	RT1 class Ib, locus M2(RT1-M2)	-2,6	0,00
NM_001109890	Protein tyrosine phosphatase, receptor type, C(Ptprc)	-2,47	0,00
NM_012705	CD4 molecule(CD4)	-2,43	0,00
NM_183051	RT1 class II, locus DOa(RT1-DOa)	-2,29	0,04
NM_001047103	Cell adhesion molecule 3(Cadm3)	-2,14	0,01
NM_001033998	Integrin subunit alpha L(Itgal)	-2,02	0,00
NM_001271086	Sialophorin(Spn)	-1,97	0,00
NM_012711	Integrin subunit alpha M(Itgam)	-1,95	0,01
NM_001100841	Laminin subunit beta 3(Lamb3)	-1,95	0,03
NM_001037780	Integrin subunit beta 2(Itgb2)	-1,88	0,01
NM_001107777	Sialic acid binding Ig like lectin 1(Siglec1)	-1,88	0,01
NM_001008884	RT1 class II, locus Db1(RT1-Db1)	-1,84	0,01
NM_001130583	Parvin, gamma(Parvg)	-1,78	0,01
NM_001008846	RT1 class II, locus DOb(RT1-DOb)	-1,77	0,02
NM_001008831	RT1 class II, locus Ba(RT1-Ba)	-1,7	0,01
NM_001008847	RT1 class II, locus Da(RT1-Da)	-1,69	0,01
NM_001013230	Selectin P ligand(Selplg)	-1,62	0,02
NM_001107737	Integrin subunit alpha 4(Itga4)	-1,61	0,02
NM_138879	Selectin E(Sele)	-1,50	0,04
NM_013171	Integrin subunit beta 7(Itgb7)	-1,50	0,04
NM_053304	Collagen type I alpha 1 chain(Col1a1)	-1,41	0,04
NM_001037336	Leucine rich repeat containing 4(Lrrc4)	1,49	0,03
NM_001108726	Integrin subunit beta 8(Itgb8)	1,49	0,03
NM_001013062	Thrombospondin 1(Thbs1)	1,53	0,02
NM_017345	L1 cell adhesion molecule(L1cam)	1,64	0,02
NM_017198	p21 (RAC1) activated kinase 1(Pak1)	1,64	0,01
NM_013117	Collagen type XI alpha 1 chain(Col11a1)	2,00	0,00
NM_031699	Claudin 1(Cldn1)	2,29	0,00
NM_001033062	Claudin 23(Cldn23)	2,36	0,01
NM_001008832	RT1 class I, locus1(RT1-CE1)	3,23	0,00
NM_012929	Collagen type II alpha 1 chain(Col2a1)	3,45	0,00
NM_001033986	RT1 class I, locus CE5(RT1-CE5)	3,69	0,00
NM_133651	Caveolin 1(Cav1)	6,74	0,00

Supplementary table 4. List of the top Chemokine Signaling Pathway and Leukocyte transendothelial migration dysregulated transcripts in glomeruli of MRS1754-treated DN rats. Downregulated and upregulated genes are represented in red and blue respectively.

GENBANK ACCESSION	GENE NAME	logFC	*p*-value
NM_001008513	C-C motif chemokine ligand 21(Ccl21)	-6,1	0,02
NM_013025	C-C motif chemokine ligand 3(Ccl3)	-3,31	0,00
NM_145672	C-X-C motif chemokine ligand 9(Cxcl9)	-3,08	0,01
NM_001127304	Neutrophil cytosolic factor 4(Ncf4)	-2,59	0,00
NM_012759	vav guanine nucleotide exchange factor 1(Vav1)	-2,42	0,00
NM_024145	FGR proto-oncogene, Src family tyrosine kinase(Fgr)	-2,26	0,00
NM_012713, NM_001172305	Protein kinase C, beta(Prkcb)	-2,23	0,00
NM_133534	C-X3-C motif chemokine receptor 1(Cx3cr1)	-2,22	0,00
NM_020542	C-C motif chemokine receptor 1(Ccr1)	-2,07	0,00
NM_053396	Adenylate cyclase 7(Adcy7)	-2,07	0,00
NM_001004202	Chemokine (C-C motif) ligand 6(Ccl6)	-2,01	0,00
NM_001191923	Phosphoinositide-3-kinase, regulatory subunit 5(Pik3r5)	-1,97	0,01
NM_053960	Chemokine (C-C motif) receptor 5(Ccr5)	-1,89	0,01
NM_013185	HCK proto-oncogene, Src family tyrosine kinase(Hck)	-1,77	0,01
NM_001108978	Phosphatidylinositol-4,5-bisphosphate 3-kinase, catalytic subunit delta(Pik3cd)	-1,67	0,01
NM_001008384	ras-related C3 botulinum toxin substrate 2 (rho family, small GTP binding protein Rac2)(Rac2)	-1,63	0,02
NM_053478	Phospholipase C, beta 2(Plcb2)	-1,59	0,03
NM_001108248	Wiskott-Aldrich syndrome(Was)	-1,41	0,04
NM_031005	Actinin, alpha 1(Actn1)	1,31	0,05
NM_001107239	Adenylate cyclase 1(Adcy1)	1,36	0,04
NM_017198	p21 (RAC1) activated kinase 1(Pak1)	1,64	0,01
NM_031007	Adenylate cyclase 2(Adcy2)	2,04	0,01
NM_031055	Matrix metallopeptidase 9(Mmp9)	2,39	0,00
NM_012605	Myosin light chain, phosphorylatable, fast skeletal muscle(Mylpf)	5,95	0,02
NM_001191065	SHC adaptor protein 4(Shc4)	6,93	0,00
