# Supplementary material for: Intraglomerular Monocyte/Macrophage Infiltration and Macrophage–Myofibroblast Transition during Diabetic Nephropathy Is Regulated by the A2B Adenosine Receptor
Source: Cells. 2020 Apr 23;9(4):1051. doi: 10.3390/cells9041051 (PMC7226348; doi:10.3390/cells9041051)
Supplement: Supplementary file 1 [file cells-09-01051-s001.zip › Supplementary table 5.pdf]

Supplementary table 5. Flow cytometry analysis of mean percent of Macrophages (CD68+) or Myofibroblasts (α-SMA+) positives and negatives for α-SMA or CD68 markers in the glomeruli of Ctrl, DM+Veh and DM+MR1754 rats.

	CD68+ (%)		α-SMA+(%)	
**Group**	**α-SMA+**	**α-SMA-**	**CD68+**	**CD68-**
Ctrl	77,628	22,371	99,063	0,936
DM+Veh	97,879	2,120	99,282	0,717
DM+MRS1754	71,259	28,740	99,174	0,825
